# Global potential for the growth of fresh groundwater resources with large beach nourishments

**DOI:** 10.1038/s41598-019-48382-z

**Published:** 2019-08-28

**Authors:** S. Huizer, A. P. Luijendijk, M. F. P. Bierkens, G. H. P. Oude Essink

**Affiliations:** 1 Department of Physical Geography, Utrecht University, Utrecht, Netherlands; 2 Arcadis, PO Box 264, Arnhem, 6800 AG Netherlands; 3 Faculty of Civil Engineering and Geosciences, Department of Hydraulic Engineering, Delft University of Technology, Delft, Netherlands; 4 Department of Hydraulic Engineering, Deltares, Delft, Netherlands; 5 Department of Subsurface and Groundwater Systems, Deltares, Utrecht, Netherlands

**Keywords:** Hydrology, Environmental impact, Hydrology

## Abstract

Whether a coastal area is suitable for beach nourishments and can induce a growth in fresh groundwater resources depends on the appropriateness of the intended site for beach nourishments, and the attainable growth in fresh groundwater resources. In this study we presume that all eroding sandy beaches are suitable for large beach nourishments, and focus on the impact of these nourishments on fresh groundwater in various coastal settings. The growth in fresh groundwater resources – as a consequence of the construction of a beach nourishment – was quantified with 2-D variable-density groundwater models, for a global range in geological parameters and hydrological processes. Our simulation results suggest that large beach nourishments will likely lead to a (temporary) increase of fresh groundwater resources in most settings. However, for a substantial growth in fresh groundwater, the coastal site should receive sufficient groundwater recharge, consist of sediment with a low to medium hydraulic conductivity, and be subject to a limited number of land-surface inundations. Our global analysis shows that 17% of shorelines may consist of erosive sandy beaches, and of these sites 50% have a high potential suitability. This shows a considerable potential worldwide to combine coastal protection with an increase in fresh groundwater resources.

## Introduction

Millions of people reside in coastal areas that are vulnerable to coastal flooding, and projections show that the (global) population in these areas will increase significantly in the coming decades^1^. Sea-level rise will likely lead to an increase in the frequency and severity of coastal flooding, in particular in tropical areas, and therefore exacerbate flood risk^2,3^. Other effects of sea-level rise are deteriorating coastal wetlands, beach erosion, seawater intrusion, and impeded drainage^4^. Without adaptation this will lead to large losses of habitable and agricultural land, as well as increases in seawater intrusion in surface waters and coastal aquifers especially in combination with the human-induced subsidence that occurs in many coastal areas^5–7^. Thus, these vulnerable coastal areas require appropriate adaptation responses to manage the flood risk and reduce negative flood consequences^4,8,9^.

For open (sand, gravel or mixed) beaches one of the potential adaptation responses are beach nourishments (also called replenishments), which have been widely and successfully applied as a counter measure for coastal recession^10–16^. Until recently, these nourishments have primarily been applied in relatively small volumes, often on a regular basis, and close to the shoreline^17^. However, in 2011 a pilot project with a large concentrated beach nourishment of ca. 21 million m^3^ of sand, called the Sand Engine (also named Sand Motor), was realized in the Netherlands^18^. The replenished sand was designed to be largely distributed along the coast and into the dunes by natural forces (e.g. waves, currents, and wind). This ‘Building with Nature’ approach is anticipated to provide a more ecologically sustainable alternative than other coastal protection approaches, in particular with regard to the current practice of frequent small-scale shoreface and beach nourishments^19^. When proven that this pilot project is successful, it may become a more widespread solution for open coasts^20^.

Beach nourishments – in particular large and concentrated nourishments – can simultaneously lead to an increase of local fresh groundwater resources in coastal areas^21^. However, the potential effect of such nourishments has only been comprehensively investigated for the Sand Engine in the Netherlands. The growth of fresh groundwater resources – as a consequence of the construction of a beach nourishment – will strongly depend on the local conditions at a site. For instance, Huizer *et al*.^22^ showed that the Sand Engine in the Netherlands is particularly vulnerable to land-surface inundations and seawater intrusion. In addition, this vulnerability to land-surface inundations will likely increase over time, because of the inevitable geomorphological changes of the beach nourishment within the’Building with Nature’ approach. Based on this local study of Huizer *et al*.^22^ one can deduce that changes in beach slope, groundwater recharge and tidal ranges will likely result in substantial deviations in the increase of fresh groundwater resources at a coastal site. Taking these importance factors into account, this study aims to provide a first estimate of the effects of concentrated large-scale beach nourishments on fresh groundwater resources at coastal areas around the world.

First, the global suitability of coasts for beach nourishments is assessed, based on an evaluation of the presence of an open beach, coastal erosion, and (if available) previous nourishments. Secondly, for the potentially suitable coastal areas, the most essential (geological) properties and (hydrological) processes that affect fresh groundwater resources are analysed: groundwater recharge, geological properties (i.e. hydraulic conductivity and specific yield), slope nourished beach, tides, coastal erosion (morphological change). The variability of each property or process is estimated with global datasets, and the effects on fresh groundwater resources is assessed and demonstrated with conceptual 2-D model simulations. In this study we focus on the current conditions of sandy beaches, and have excluded future changes in sea-level or groundwater recharge due to climate change. While changes in both of these processes will affect coastal fresh groundwater resources^21^, the global variability and uncertainty in the predicted changes render it too complex for this conceptual study. Finally, based on the results of the simulations, the implications for sandy shorelines worldwide are discussed.

## Results

For each of the top six listed processes or properties (see Method section), the simulated (1) initial condition or equilibrium state, and (2) impact of the beach nourishment on the fresh groundwater resources (as a function of time) are described and visualized in the following paragraphs. Extensive land-surface inundations due to storm surges, tropical cyclones or tsunamis were excluded, as simulating these events is beyond the scope of this study. Nonetheless, as previously shown^22,23^, frequent to occasional inundations – especially beyond the intertidal area – can have a strong impact on the growth of the fresh groundwater volume. Therefore, it is treated as one of the most important factors with respect to the growth of coastal fresh groundwater resources in large beach nourishments and is included in the assessment of the global suitability of coasts for beach nourishments.

Every model scenario contained an addition of a 100 m wide beach nourishment at the start of the simulation period, which slowly erodes to the original setup. In all model scenarios the application of the specified nourishment leads to an enlargement of the existent fresh groundwater lens, regardless of the changes in the processes or properties. The placement of the beach nourishment results in a seaward shift of the intertidal zone, and consequently – if extensive land-surface inundations are neglected – to a wider area in which the fresh groundwater lens can advance. The simulated gradual erosion of the beach nourishment – or retreat of the shoreline – subsequently leads to a decline of the beach width over time. This in turn, ensures that the maximum growth in fresh groundwater (i.e. optimum) occurs before the end of the simulation period. Since this pattern is similar in all scenarios, the evaluation of the impact of each process or property will focus on the (relative) growth rate and its change in time.

### Groundwater recharge

**Figure 1:**
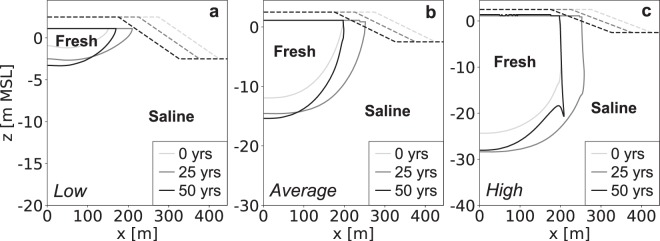
Simulated growth of the fresh groundwater lens for low (left), average (centre), and high (right) groundwater recharge rates. All images contain the fresh-salt groundwater interface of 1 g TDS L^−1^ (solid lines) and the surface elevation (dashed lines) at the start (light grey), middle (dark grey) and end (black) of the simulation period.

**Figure 2:**
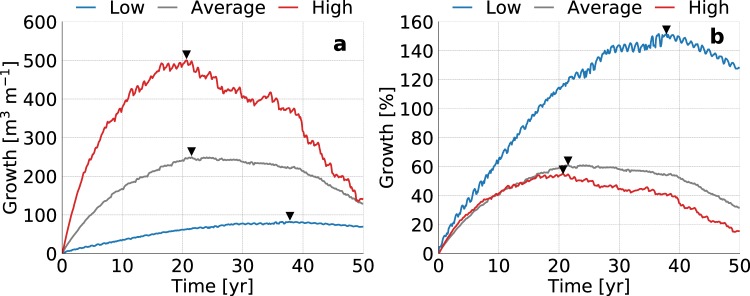
Simulated growth of fresh groundwater for low (blue line), average (dark grey line), and high (red line) estimates of the global groundwater recharge, where the black triangles point to the time with the maximum growth of fresh groundwater. The graphs represent the change in volume (left) and percentage (right) with respect to the initial conditions (i.e. equal to equilibrium state without beach nourishment).

Increases in groundwater recharge lead to higher groundwater levels, larger fresh groundwater lenses (Fig. 1), and larger absolute growth rates of the volume of fresh groundwater (Fig. 2a). However, low groundwater recharge rates result in considerably higher relative growth rates, and a longer period in which the fresh groundwater lens expands (Fig. 2b). This is because larger (absolute) growth rates result in an earlier attainment of the maximum size (i.e. equilibrium: growth equals losses) of the fresh groundwater lens. However, this is only true when the (phreatic) aquifer is thick enough to allow for a continued growth of the fresh groundwater lens. In all model simulations the thickness of the phreatic aquifer was extended accordingly to avoid an impeded growth of the fresh groundwater lens (Fig. 1c).

### Hydraulic conductivity

**Figure 3:**
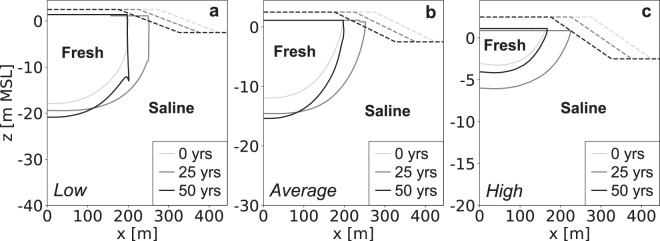
Simulated growth of the fresh groundwater lens for low (left), average (centre), and high (right) estimates of the hydraulic conductivity in sandy shores. All images contain the fresh-salt groundwater interface of 1 g TDS L^−1^ (solid lines) and the surface elevation (dashed lines) at the start (light grey), middle (dark grey) and end (black) of the simulation period.

**Figure 4:**
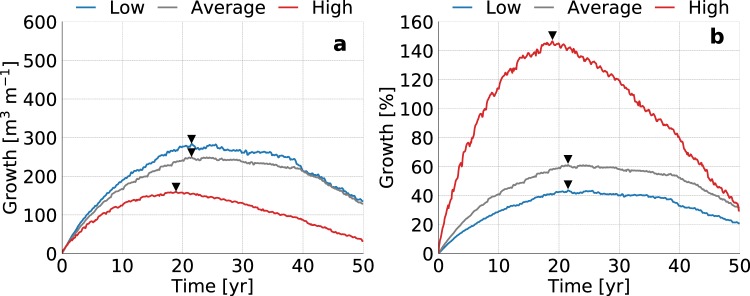
Simulated growth of fresh groundwater for low (blue line), average (dark grey line), and high (red line) estimates of the global hydraulic conductivities, where the black triangles point to the time with the maximum growth of fresh groundwater. The graphs represent the change in volume (left) and percentage (right) with respect to the initial conditions (i.e. equal to equilibrium state without beach nourishment).

Low to average hydraulic conductivities lead to the highest groundwater levels, largest fresh groundwater lenses (Fig. 3a,b), and the highest absolute growth rates of the fresh groundwater volume (Fig. 4a). However, shores with high hydraulic conductivities result in considerably higher relative growth rates (Fig. 4b). In a shore with a high hydraulic conductivity the growth remains relatively small in volume, but large in comparison with the initial volume of fresh groundwater. The period in which the volume of fresh groundwater increases also changes with the hydraulic conductivity, because shores with higher hydraulic conductivities are more strongly influenced by tidal forcing. This additionally leads to higher loss rates, and a steeper decline in the volume of fresh groundwater.

### Specific yield

**Figure 5:**
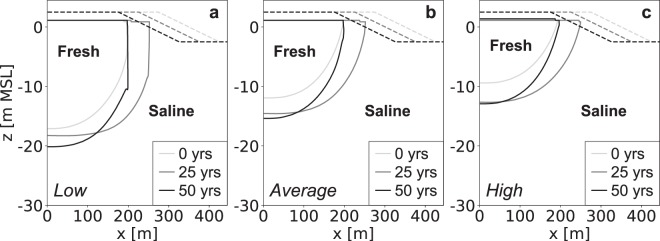
Simulated growth of the fresh groundwater lens for low (left), average (centre), and high (right) estimates of the specific yield. All images contain the fresh-salt groundwater interface of 1 g TDS L^−1^ (solid lines) and the surface elevation (dashed lines) at the start (light grey), middle (dark grey) and end (black) of the simulation period.

**Figure 6:**
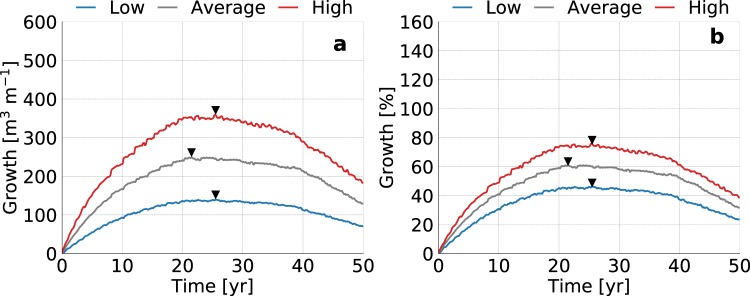
Simulated growth of fresh groundwater for low (blue line), average (dark grey line), and high (red line) estimates of the global specific yield in sandy shores, where the black triangles point to the time with the maximum growth of fresh groundwater. The graphs represent the change in volume (left) and percentage (right) with respect to the initial conditions (i.e. equal to equilibrium state without beach nourishment).

The simulations show that a smaller specific yield leads to a larger fresh groundwater lens (Fig. 5). However, as a result of the reduced storage capacity a smaller specific yield – despite the larger fresh groundwater lens – results in a decrease of the absolute growth of the fresh groundwater volume (Fig. 6a). The relative growth rates for low, average, and high values of the specific yield confirm that sites with larger specific yields result in a higher growth rate (Fig. 6b). Therefore, shores with a higher specific yield are generate the largest growth in fresh groundwater resources.

### Slope nourished beach

**Figure 7:**
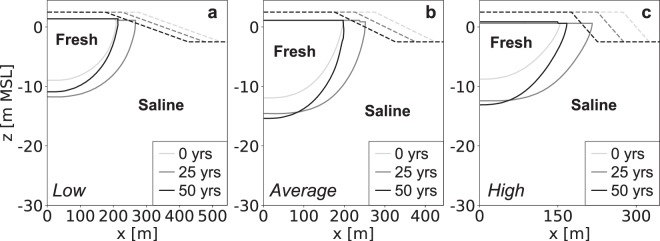
Simulated growth of the fresh groundwater lens for low (left), average (centre), and high (right) estimates of the slope of the nourished beach. All images show the fresh-salt groundwater interface of 1 g TDS L^−1^ (solid lines) and surface elevation (dashed lines) at the start (light grey), middle (dark grey) and end (black) of the simulation period.

**Figure 8:**
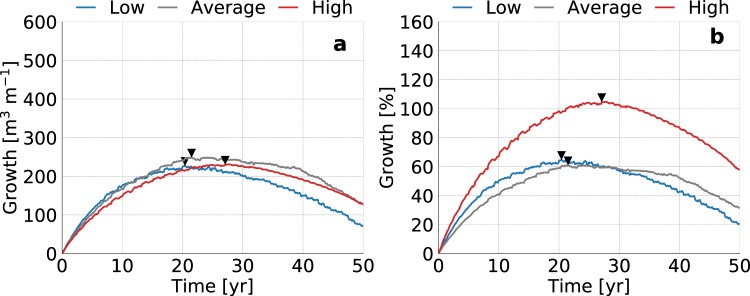
Simulated growth of fresh groundwater for low (blue line), average (dark grey line), and high (red line) estimates of the global slope of the nourished beach in sandy shores, where the black triangles point to the time with the maximum growth of fresh groundwater. The graphs represent the change in volume (left) and percentage (right) with respect to the initial conditions (i.e. equal to equilibrium state without beach nourishment).

Increases in the slope of the nourished beach result in narrower intertidal zones, which lead to a reduction of the area in which seawater infiltrates (within the same tidal range). The decrease in infiltration leads to lower groundwater levels (Fig. 7), and subsequently the maximum (potential) depth of the fresh-salt groundwater interface becomes smaller. However, the relation between the slope of the nourished beach and the groundwater level is non-linear: in beaches with lower slopes the increase in the infiltration of seawater will have a stronger impact on the fresh groundwater volume at some stage. This is the reason for the (slightly) larger fresh groundwater lens in the simulation with an average slope of the nourished beach (Fig. 7b). In addition, a narrower intertidal zone and decrease in infiltration of seawater leads to a longer period of growth (Fig. 8a), with a substantially higher relative growth rate (Fig. 8b).

### Tidal range

**Figure 9:**
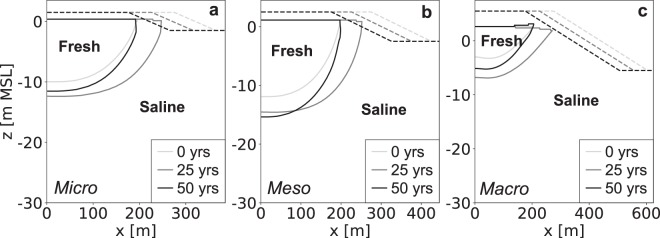
Simulated growth of the fresh groundwater lens for micro (left), meso (centre), and macro (right) tidal ranges. All images show the fresh-salt groundwater interface of 1 g TDS L^−1s^ (solid lines) and the surface elevation (dashed lines) at the start (light grey), middle (dark grey) and end (black) of the simulation period.

**Figure 10:**
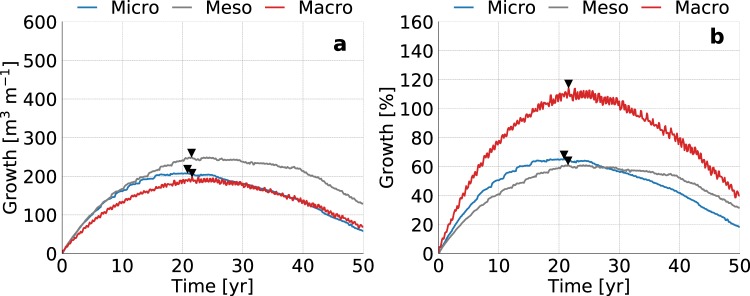
Simulated growth of fresh groundwater for micro (blue line), meso (dark grey line), and macro (red line) tidal ranges, where the black triangles point to the time with the maximum growth of fresh groundwater. The graphs represent the change in volume (left) and percentage (right) with respect to the initial conditions.

Sites with intermediate tidal ranges attain the largest fresh groundwater lens (Fig. 9b) and highest absolute growth during the simulation period (Fig. 10a). This is a result of two counteracting processes that are related to the increase in (net) seawater intrusion for sites with larger tidal ranges. Increases in the infiltration of seawater in the intertidal zone leads (1) to higher groundwater levels and therefore a larger potential depth of the fresh-salt groundwater interface, but (2) also result in a salinization of fresh groundwater resources within and near the intertidal zone. The optimum with respect to fresh groundwater resources, occurs therefore in intermediate tidal ranges.

### Coastal erosion

**Figure 11:**
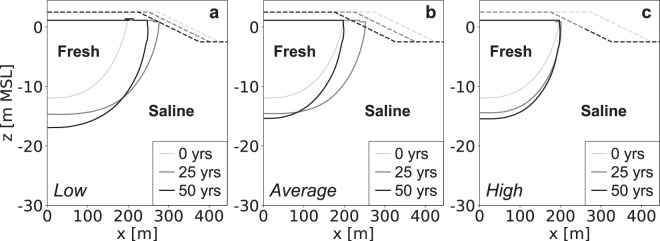
Simulated growth of the fresh groundwater lens for low (left), average (centre), and high (right) estimates of coastal erosion. All images show the fresh-salt groundwater interface of 1 g TDS L^−1^ (solid lines) and the surface elevation (dashed lines) at the start (light grey), middle (dark grey) and end (black) of the simulation period.

**Figure 12:**
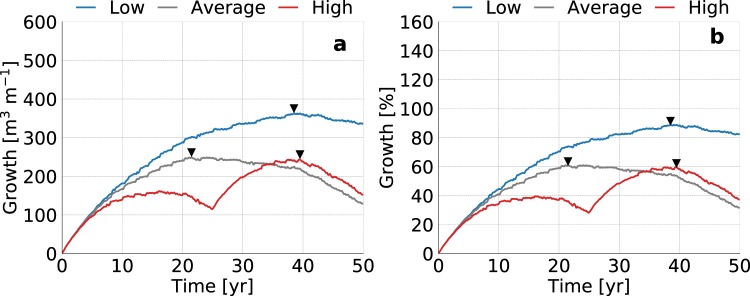
Simulated growth of fresh groundwater for low (blue line), average (dark grey line), and high (red line) estimates of the global coastal erosion in sandy shores, where the black triangles point to the time with the maximum growth of fresh groundwater. The graphs represent the change in volume (left) and percentage (right) with respect to the initial conditions (i.e. equal to equilibrium state without beach nourishment).

The development of the fresh groundwater lens is initially equal, and only starts to diverge when the varying erosion rates start to limit the growth rates or cause a loss in fresh groundwater (Fig. 11a,b). Obviously, lower erosion rates result in larger fresh groundwater lens and the largest growth rates, reaching a maximum volume of fresh groundwater after 16 (after first nourishment), 21.5, and 38.5 years respectively (Fig. 12). This shows that in most previous scenario’s erosion is an important limiting factor the growth of fresh groundwater resources. It should be noted that in the simulation with a high erosion rate included two beach nourishments, one at the start and one after 25 years, to prevent an erosion beyond the initial shoreline. This shows that two consecutive nourishments lead to a larger growth of the fresh groundwater lens.

### Global suitability

In all model scenarios, the addition of a beach nourishment to the coast results in a (temporary) increase of fresh groundwater resources. The growth rate is highest in the first years after the nourishment is added to the site. But over time the (simulated) retreat of the shoreline leads to a decrease of the growth rate, which eventually turns in to a decrease in the attained fresh groundwater volume. This pattern is to be expected, and likely for most cases where a beach is widened. Over a wide range of processes and properties, the simulations show that more groundwater recharge, smaller hydraulic conductivities, a larger specific yield (i.e. storage capacity), and less coastal erosion, in particular lead to a large growth of the fresh groundwater volume. However, it should be noted that larger hydraulic conductivities and smaller groundwater recharge rates bring about larger relative increases in fresh groundwater resources (i.e. relative to the initial condition or equilibrium state). Changes in the slope of the nourished beach, and tidal dynamics have a relatively small impact on the attained growth in fresh groundwater.

As the simulation of extensive land-surface inundations due to storm surges, tropical cyclones or tsunamis were beyond the scope of this study, the impact of such events on fresh groundwater resources could not be directly assessed from the model simulations in this study. However, previous research has shown that extensive land-surface inundations will likely have a substantial impact on coastal fresh groundwater in sandy beaches^22,23^. Dependent on the extent and duration of the inundation, a portion or all of the accumulated fresh groundwater could be lost due to salinization. In addition, the design of the beach nourishment (e.g. crest elevation) will also determine the susceptibility of a site for land-surface inundations. Therefore, we argue that extensive land-surface inundations is one of the decisive factors in the growth of fresh groundwater resources by large beach nourishments at a coastal site.

**Figure 13:**
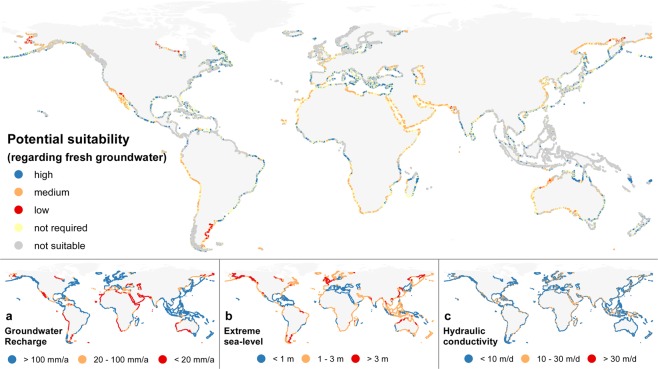
Global map of the potential suitability (high, medium, low) of eroding sandy shores for the growth of coastal fresh groundwater resources by large beach nourishments, based on the combination of global suitability maps for (**a**) groundwater recharge, (**b**) extreme sea-levels, (**c**) and coastal erosion (see Table 1).

With respect to the hydraulic conductivity, the global permeability map of Huscroft *et al*.^24^ indicates that most of the unconsolidated sediment has a hydraulic conductivity smaller than 25 m d^−1^. Therefore, the hydraulic conductivity in most sandy beaches is likely low to average, and similarly we expect that most sandy beaches will have a specific yield close to the average (see Method section). While both hydraulic conductivity and specific yield remain important factors for the growth of the fresh groundwater volume, we argue that (on a global scale) the most decisive factors for the growth of fresh groundwater resources by large beach nourishments are groundwater recharge, (extensive) land-surface inundations, and coastal erosion. Based on this assertion we created a global map that depicts the potential suitability of sandy shores for the growth of coastal fresh groundwater resources by large beach nourishments (Fig. 13). The potential suitability was defined as the extent to which the characteristics of a coastal site can potentially lead to a growth of the fresh groundwater volume, and simultaneously reduce the likelihood of losses due to extensive inundations or coastal erosion.

Focusing only on the eroding sandy shores – 17% of all analysed coastal sites, the subsequent potential suitability was assessed with global maps of (a) groundwater recharge^25^, (b) height of extreme sea-levels with a return period of 100 years^26^, and (c) coastal erosion^27^. The height of extreme sea-levels was used as an indication of the risk of extensive land-surface inundations at a coastal site on a global scale. Three classes of increasing suitability – low, medium, and high – were defined, based on the combination of global suitability maps for (a) groundwater recharge, (b) extreme sea-levels, and (c) coastal erosion (Fig. 13). To each of these global maps weights were assigned of −2 or −1 (Low), 0 (Medium), and +1 or +2 (High), which are shown for all classes in brackets in Table 1. The weights were assigned in accordance with the results of the sensitivity analysis. The sum of the weights (a + b + c) were used to define the potential suitability: high for weights equal or larger than 3, medium for weights between −3 and 3, and low for weights equal or smaller than −3. Coastal erosion was assigned a 50% lower weight, in accordance with the simulation results and the expected deviation in the impact on the growth of fresh groundwater resources.

**Table 1:** Weights assigned to (a) groundwater recharge, (b) extreme sea-levels, and (c) coastal erosion, which combined result in the suitability (low, medium, high) of eroding sandy shores for a growth of fresh groundwater resources by large beach nourishments.

Suitability Class	a: Groundwater recharge	b: Extreme sea-level	c: Coastal erosion
High (≥3)	>100 mm yr^−1^ (+2)	<1 m MSL (+2)	−1 to −0.5 m yr^−1^ (+1)
Medium (−3 to 3)	20–100 mm yr^−1^ (0)	1–3 m MSL (0)	−3 to −1 m yr^−1^ (0)
Low (≤−3)	<20 mm yr^−1^ (−2)	>3 m MSL (−2)	<−3 m yr^−1^ (−1)

The potential suitability map shows that on 17% of the analysed coastal sites large beach nourishments can lead to a growth in fresh groundwater resources, and of these sites 50% have a high potential suitability, 46% have a medium potential suitability, and 4% have a low potential suitability. This analysis suggests that on most of the eroding sandy sites, large beach nourishments can lead to growth of the (local) fresh groundwater volume.

## Discussion

The question whether a coastal site is suitable for large beach nourishments and will subsequently lead to an increase of fresh groundwater resources is complex. In a first attempt to obtain answers with regard to the increase in the volume of fresh groundwater, the assumption was made that all eroding sandy beaches are technically suitable (see Method section). While this may be true for most eroding sandy beaches, it is important to note that detailed local studies should be conducted to answer this question for specific coastal sites.

The conceptual 2-D model simulations showed that – on a global scale – groundwater recharge and coastal erosion are the most decisive factors for the growth in fresh groundwater by large beach nourishments. However, this study focused on the current (climate) conditions (i.e. excluded sea-level rise and other changes in climate) and did not directly analyse the effects of extensive land-surface inundations due to storm surges, tropical cyclones or tsunamis. Previous research has shown that both processes will likely reduce or limit the growth of coastal fresh groundwater in sandy beaches and in large beach nourishments^22,23^. Therefore, when included in the simulations, the growth in fresh groundwater resources could likely be smaller than projected in our study. Because the simulations were primarily intended to show the sensitivity of the growth in fresh groundwater resources to changes in (hydrological) processes and (geological) properties of sandy beaches, this does not affect the reliability of the results. It only means the analysis is not comprehensive, as it is not possible to weigh the impact of sea-level rise and extensive land-surface inundations directly with groundwater recharge or coastal erosion.

Another deficiency of the adopted methodology lies in the simplified 2-D model setup, because real sandy beaches exhibit 3-D variability in for instance surface elevation, geology, groundwater and surface water interaction, and aquifer thickness. For instance, most beaches exhibit heterogeneity in sediment grain size^28^ and therefore in hydraulic conductivity, which can lead to substantial changes in the fresh – salt groundwater distribution in the coastal aquifer. Besides space, also in time a coastal site may experience large fluctuations of the surface elevation, inundated area, and (annual) groundwater recharge than were included in the model scenarios. For example, beach slopes can change significantly due to varying concentrations of suspended sediment or seasonal differences in coastal forcing (e.g. erosion in winter, accretion in summer). Therefore, while in general the conclusions are probably true for most sandy shores, (local) variations in space and time can lead to substantially different outcomes in terms of growth in fresh groundwater resources.

It is also important to note that the model setup excluded a flow of (fresh) groundwater across the inland model boundary (i.e. no-flow boundary). This was to minimize the (external) impact on the development of the fresh groundwater lens. However, most sites in the world will likely have a flow of fresh groundwater towards the sea, and most islands in the world are wider or not shaped as strip-island as implemented in the model simulations. An inflow of fresh groundwater or an increase of the island width will both increase the growth of fresh groundwater resources, resulting a higher growth rate. In addition, previous studies have shown that a decrease in fresh groundwater flow at the inland boundary increases the effect of tidal forcing, leading to larger salt groundwater plumes or tidal circulation^29,30^. Therefore – as a consequence of the adopted model setup – tidal forcing becomes the dominant force in our model simulations. Tidal circulations cause the fresh to salt groundwater interface to expand, which results in relatively large mixing zones. In real-world beaches with similar conditions the growth in fresh groundwater resources would therefore likely larger than indicated in the simulations.

## Conclusion

In many coastal sites the application of a beach nourishment will likely lead to a (temporary) increase of fresh groundwater resources. To improve the likelihood of a (substantial) growth of the volume of fresh groundwater, a coastal site should get sufficient groundwater recharge, preferable have a low to medium hydraulic conductivity, and have limited number of extensive land-surface inundations. However, it must be noted that a lower hydraulic conductivity also leads to a lower extraction rate. Coastal sites with high erosion rates will result in a lower maximum growth of fresh groundwater resources and may be more vulnerable to land-surface inundations. A global analysis revealed that an estimated 17% of the coasts consists of eroding sandy shores, which may be suitable for large-scale beach nourishments^27^. Of these eroding sandy shores 50% have a high suitability with regard to the growth of fresh groundwater resources by large beach nourishments. However, these results provide only a first assessment of the possibility of an increase of the fresh groundwater resources by beach nourishments. For a detailed and more reliable prediction of the growth of the fresh groundwater volume on a site, additional site-specific research should be conducted.

## Method

### Suitable locations for large-scale beach nourishments

Recent research^27^ suggests that 31% of the ice-free shorelines in the world consist of sandy beaches – including quartz and carbonate sands, and gravel – and that 24% of these sandy beaches were subjected to coastal erosion of more than 0.50 m per year in the period 1984 to 2016. These eroding coastlines may satisfy the primary conditions for (large-scale) beach nourishments: a coastal environment that conforms to (beach) nourishments, and local communities or stakeholders that desire and benefit from coastal protection (e.g. prevent loss of land). Of course, beach nourishments could also be implemented to widen recreational beaches or to create new beaches in coastal areas where none existed before. However, in this study we focus on sites that suffer from beach erosion, in order to comply with the ‘Building with Nature’ philosophy^19^.

Whether beach nourishments are applied in coastal areas, is dependent on numerous factors and circumstances. First the desired adaptation strategy: coastal protection, accommodate, (re)claiming land from the sea, limited intervention (“passive retreat”), non-intervention, managed retreat, or ecosystem conservation^31^. Only if a coastal manager opts for coastal protection – the most common adaptation option – and has the knowledge and means to execute this strategy, nourishments are one of the possibilities. Overall, the options for the coastal protection of the shoreline position vary between hard structures (e.g. groins and seawalls) and soft engineering (i.e. nourishments). Environmental concerns and inefficiencies have led to a historical shift from predominantly hard to soft engineering solutions^17,32^, in particular if the beach serves multiple vital purposes.

The suitability of coastal areas for beach nourishments is complex and should be assessed with detailed local studies, which lies beyond the scope of this study. However, to provide some insight, we summarized the most important considerations for (large) beach nourishments in Table 2. In this study we focus on all (eroding) sandy beaches worldwide and assume that these beaches are technically suitable for large-scale beach nourishments.

**Table 2:** Important (technical) considerations for sustainable (large) beach nourishments.

Nr	Attribute	Cons iderations for successful beach nourishments
1	Aeolian transport	Aeolian transport of the nourished sand is highly dependent on the median grain size, grain-size distribution, and the spatial variability of sand properties (e.g. presence of shell fragments)^45^.
2	Erosion rate	Generally, nourished beaches erode at least as fast as the original or pre-nourished coastline, and often even faster^46^.
3	Natural habitat	Beach nourishments are a more ecologically sound alternative to hard engineered structures, but nourishments can also cause damage to habitat and biota. Negative effects should be mitigated, e.g. match pre-nourished conditions^47,48^.
4	Developed shores	Beach nourishments are predominantly applied on marine, developed, sandy beaches, where the beach also serves as protection of coastal infrastructure and as recreational site^46^.
5	Sediment grain-size	The grain-size of the nourished sediment is generally slightly coarser than the original sediment^49,50^.
6	Sediment source	The sediment source (dredge site or terrestrial source) that can provide the required composition and quantity of sediment should be relatively close to site to be cost-effective^51^.
7	Sediment transport	The redistribution of the nourished sediment is a complex process, which is dependent on coastal forcing, coastal characteristics (e.g. profile), and the nourishment shape and dimensions^52^.

### Processes and properties underlying fresh groundwater development

**Figure 14:**
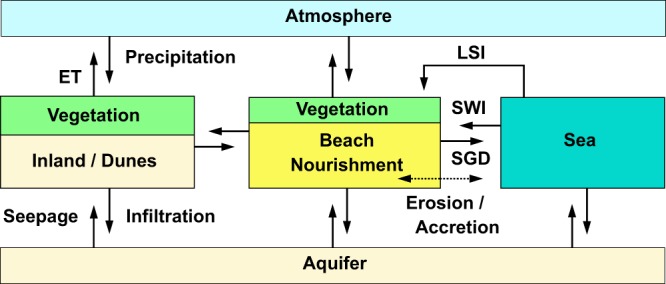
Hydrological, morphological, and ecological processes (arrows) that affect the growth of fresh groundwater resources in a (large) beach nourishment (LSI = land-surface inundations, ET = evapotranspiration, SGD = submarine groundwater discharge, and SWI = seawater intrusion).

Huizer *et al*.^21^ showed that fresh groundwater resources can grow substantially as a consequence of the construction of a mega-scale beach nourishment. However, whether this growth will occur at a coastal site is dependent on the inflow rate, outflow rate, storage capacity, and losses of fresh groundwater to the deeper coastal aquifer. Recent research^22,23^ demonstrated the significance of these dynamic processes to the development of fresh groundwater resources, and illustrated for example the considerable impact of wave exposure and storm surges. The combined hydrological, morphological, and ecological processes (arrows) that affect the growth of fresh groundwater in a (large) beach nourishment are illustrated in Fig. 14, where for simplicity anthropogenic effects such as groundwater abstractions were neglected.

It is important to note that these processes can affect one another, because each process can alter the groundwater head, hydraulic gradient, and the groundwater flow. For example, when land-surface inundations (LSI) lead to a substantial increase of the groundwater head, this in turn can lead to a larger evapotranspiration rate or more submarine groundwater discharge (SGD). Or, when a storm surge leads to extensive coastal flooding, this often also results in coastal erosion and can have a detrimental effect on flooded dune vegetation. Changes in surface elevation or loss of dune vegetation can in turn also alter the evapotranspiration rates.

While the hydrological processes shown in Fig. 14 largely drive the (positive or negative) growth of fresh groundwater resources, the geological properties of the nourished sediment and the (local) coastal aquifer determine the infiltration rate, storage capacity, and groundwater flow rates. For example, the grain size and sorting of the nourished and original sediment will strongly determine the hydraulic conductivity and (effective) porosity of the coastal aquifer. Similarly, the geological properties will affect the (maximum) flow rate and flow direction of the groundwater fluxes, as for instance larger hydraulic conductivity will result in larger infiltration rates.

To assess the impact of hydrological processes and various other (geological) properties, we have examined and described the effects of a selection of the most important properties and processes on fresh groundwater resources (Table 3). For each of the listed (geological) properties and (hydrological) processes, the global range of variability of the particular property or process is characterized with low, average and high estimates. In addition, these low, average and high estimates are implemented in the model simulations, where the ‘average’ column represents the reference model. Clearly this selection is not comprehensive, and other properties or processes such as inland groundwater flow, geological heterogeneity, and sea-level rise will affect the built-up of fresh groundwater resources.

**Table 3:** Global variation in a selection of (hydrological) processes and (geological) properties of sandy beaches.

Nr	Process/Property	Global variation	Low	Average	High	Source(s)
1	Groundwater recharge	Very low (<2 mm yr^−1^) to very high (>300 mm yr^−1^): highest in humid tropics, lowest in dry subtropics and arctic regions.	50 mm yr^−1^	200 mm yr^−1^	500 mm yr^−1^	Döll & Fiedler^25^; Wada *et al*.^53^
2	Hydraulic conductivity	Conductivity of coarse grained unconsolidated sediment varies from 0.7 m d^−1^ to 170 m d^−1^.	10 m d^−1^	20 m d^−1^	50 m d^−1^	Huscroft *et al*.^24^; Wilson *et al*.^28^
3	Specific yield	Storage in unconsolidated sediment varies between 0.11 and 0.36.	0.10	0.20	0.30	Gleeson *et al*.^54^; De Graaf *et al*.^55^
4	Slope nourished beach	Beach slope variability for sandy beaches varies between 1:5 to 1:80 (median 1:24).	1: 10	1: 30	1: 50	McLachlan & Dorvlo^56^
5	Tidal range	Global tidal ranges: micro (<2 m), meso (2 to 4 m), to macro (>4 m).	0–2 m	2–4 m	4–5 m	PSMSL dataset^57^
6	Coastal erosion	Beach erosion or shoreline retreat range from less than 1 m per year to several meters due to one storm surge event.	1 m yr^−1^	2 m yr^−1^	4 m yr^−1^	Anthony^58^
7	Storm surge /Wave exposure	Extreme sea levels – caused by high tides, storm surges/hurricanes – from <0.2 m to >5 m (return period of 100 years).	1 m	3 m	5 m	Muis *et al*.^26^

The impact of each factor on fresh groundwater resources was evaluated with common estimates of low, average and high values within the noted limits.

### Model simulations

Besides storm surges and wave exposures, the effect of each (geological) property or (hydrological) process in Table 3 on the growth of fresh groundwater resources – in a sandy coast with a concentrated large-scale beach nourishment – was assessed with conceptual 2-D model simulations. The model simulations were executed with the computer code SEAWAT^33^ to simulate variable-density groundwater flow and coupled salt transport. The model scenarios were constructed, executed and processed with the Python package FloPy^34^, starting with a reference model that consists of a 2-D approximation of the average global sandy beach.

**Figure 15:**
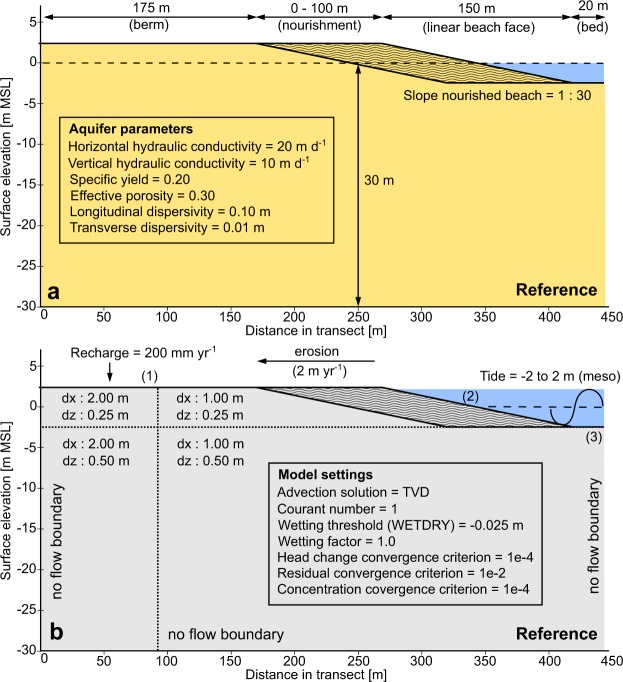
Details of the reference model: model domain, boundary conditions (**b**), aquifer parameters (**a**), model settings (**b**), recharge rate (**b**), slope nourished beach (**a**), tidal range (**b**), and erosion rate (**b**). All of the top six listed processes or properties are equal to the ‘average’ column in Table 3. The beach nourishment is indicated with the wave pattern.

The 2-D reference model (Fig. 15) was discretized into 400 columns with horizontal cell sizes of 1 to 2 m, and 75 layers with thicknesses of 0.25 to 0.50 m. In each model scenario, the smallest cell sizes were positioned in the area close to the (intended) beach nourishment and sea boundary. The design of the model domain and geometry was based on designs from previous 2-D numerical modelling studies on sandy beaches^30,35–39^. Similar to the design in these studies, the model comprises three sections: (1) constant berm height, (2) linear beach face, (3) constant bed level (Fig. 15). In all model scenarios the berm elevation was defined above the highest prescribed sea-level – and accordingly the width of the linear beach face was adapted – to avoid an inundation of the whole model domain due to tidal dynamics.

All model boundaries – besides the upper boundary – are defined as no-flow, to enable an unconstrained development of the fresh groundwater lens and fresh-salt groundwater mixing zone. Consequently, the reference model is in effect an elongated or strip island model with a width of 500 m and an aquifer thickness of 30 m. This is relatively small in comparison with real-world islands, but this was a deliberate choice to amplify the impact of coastal processes on the growth of fresh groundwater resources in and near a (large) beach nourishment, and to cut-down the overall simulation time. As the relationship between the island width and the availability of fresh groundwater is generally non-linear^36,40^, a wider island would even more lead to a larger fresh groundwater lens and a significantly smaller vulnerability to seawater intrusion.

For the simulation of the sea boundary the same method as in Huizer *et al*. (2017, 2018) was adopted, and for more information on the methodology we refer to these studies. In summary, the boundary was modelled as a time-variable specified head and concentration, equal to “General Head (head-dependent) Boundaries and Drains” as described by Mulligan *et al*.^41^. Wave setup and run-up were estimated with the parametrization for setup on dissipative sites of Stockdon *et al*.^42^.

The reference model was used a starting point for all model scenarios. For each scenario only the relevant model parameters were adapted in accordance with Table 3. First, all model scenarios were (repeatedly) simulated for a period of 50 years – without the intended beach nourishment – until a (dynamic) steady-state condition was reached. Steady state was defined in terms of the change in the volume of fresh, brackish and saline groundwater in the model domain, and attained when the changes oscillate around an equilibrium. Second, a beach nourishment with a width of 100 m and a crest elevation equal to the berm height, was added to each model scenario (Fig. 15). Subsequently, each scenario was simulated for a period of 50 years to assess the impact on the volume of fresh groundwater.

### Tidal forcing

As stated in Table 3, a tidal regime with a meso tidal range (2 to 4 m) was implemented in the reference model, whereas tidal regime with micro (<2 m) and macro (>4 m) tidal ranges were included as model scenarios. Global representations of micro, meso and macro tidal regimes were obtained from the Research Quality Data Set of the University of Hawaii Sea Level Center, which is part of the Global Sea Level Observing System (GLOSS) Delayed Mode Higher Frequency dataset^43^. This dataset contains tide gauge measurements of 547 stations around the world that have received quality control. Each tide gauge station was classified into a micro, meso or macro tidal range through the calculation of the mean tidal range of each timeseries. Only measurements after 01-01-1970 were included to ensure that the calculated tidal ranges were representative for present-day conditions.

Based on the measurement period and mean tidal range of each station, three tide gauge stations were selected to represent the micro, meso and macro tidal regimes in the model simulations (Table 4). The observed tidal behaviour at these three locations was extrapolated to the period 1970 to 2020, to fill time gaps in the measurements and to obtain tide gauge data with a timestep of 10 minutes instead of one hour. For the extrapolation of the micro, meso and macro tidal regimes we used the Python package Pytides, which uses the method of harmonic constituents^44^. Sea-level rise was not incorporated in the extrapolation of the tidal regimes.

**Table 4:** Selection of tide gauge stations from the GLOSS Delayed Mode Higher Frequency dataset that signify micro, meso and macro tidal ranges.

Tide Class	GLOSS	Station	Country	Measurement Period	Mean tidal range
Micro	111	Kwajalein	Marshall Islands	1946–2014	1.01 m
Meso	284	Cuxhaven	Germany	1917–2014	2.93 m
Macro	040	Broome	Australia	1986–2015	5.51 m

### Mapping global suitability

The results of the conceptual 2-D model simulations were subsequently used to assess the effect of each (geological) property or (hydrological) process in Table 3 – besides storm surges and wave exposures – on the growth of fresh groundwater resources in a sandy coast with a (newly created) concentrated large-scale beach nourishment. For the properties or processes with the highest impact on fresh groundwater resources, the consequences of this analysis were translated and visualized to a global suitability map (Fig. 13) with help of global datasets (Table 3). This map portrays the potential growth of fresh groundwater at a coastal site as a result of the construction of a large-scale beach nourishment.

Because extensive land-surface inundations – i.e. inundations caused by storm surges, tropical cyclones or tsunamis – were excluded the simulations, the impact of such events on fresh groundwater resources could not be directly assessed from the model simulations in this study. However, based on previous research^22,23^ we have included it as one of the decisive factors in the growth of fresh groundwater resources by large beach nourishments. The impact of extensive land-surface inundations was qualitatively assessed, based on the results of the simulations with (micro, meso, and macro) tidal ranges and (low, average, and high) erosion rates. The basis of the translation was a global map with extreme sea-levels with a return period of 100 years^26^. For similar levels as observed and implemented for tidal ranges, we assume that a higher extreme sea-level substantially reduces the growth in fresh groundwater resources.

As a rough estimate of the suitability of a coastal site for beach nourishments we focused only on sandy beaches that suffer from coastal erosion. For the identification of eroding sandy shorelines the data of a recent paper^27^ was used, which developed and implemented a procedure to detect sandy beaches from satellite images of 2016 (Sentinel-2) and shoreline changes from satellite images between 1984–2016 (Landsat 5, 7 and 8). All other shorelines were excluded from the analysis – 83% of all analysed coastal sites, and are visualized as either ‘not required’ (i.e. not-eroding sandy shore: 15% of sites) or ‘not suitable’ (i.e. not sandy shore: 68% of sites) in Fig. 13. It should be noted that in some cases coastal erosion was obscured by human interventions between 1984 and 2016, and wrongly identified as ‘not suitable’. One prime example is the widespread application of beach nourishments on the Dutch coast^10^.
